# Bioactive and Biological Potential of Black Chokeberry Leaves Under the Influence of Pressurized Liquid Extraction and Microwave-Assisted Extraction

**DOI:** 10.3390/antiox13121582

**Published:** 2024-12-23

**Authors:** Maja Repajić, Ivona Elez Garofulić, Ena Cegledi, Erika Dobroslavić, Sandra Pedisić, Ksenija Durgo, Ana Huđek Turković, Jasna Mrvčić, Karla Hanousek Čiča, Verica Dragović-Uzelac

**Affiliations:** 1 University of Zagreb, Faculty of Food Technology and Biotechnology, Pierottijeva 6, 10000 Zagreb, Croatia; maja.repajic@pbf.unizg.hr (M.R.); ena.cegledi@pbf.unizg.hr (E.C.); sandra.pedisic@pbf.unizg.hr (S.P.); ksenija.durgo@pbf.unizg.hr (K.D.); ana.hudek@pbf.unizg.hr (A.H.T.); jasna.mrvcic@pbf.unizg.hr (J.M.); karla.hanousek.cica@pbf.unizg.hr (K.H.Č.); verica.dragovic-uzelac@pbf.unizg.hr (V.D.-U.); 2 University of Dubrovnik, Department of Applied Ecology, Ćira Carića 4, 20000 Dubrovnik, Croatia; erika.dobroslavic@unidu.hr

**Keywords:** *Aronia melanocarpa* L., polyphenols, antioxidant capacity, cytotoxicity, antiproliferative activity, DNA damage, antimicrobial, antifungal

## Abstract

To determine the optimal conditions of pressurized liquid extraction (PLE) and microwave-assisted extraction (MAE) of polyphenols from black chokeberry leaves (BCL), temperature, time and sample-to-solvent ratio (SSR) were varied to obtain maximum polyphenols yield. The extracts were analyzed for total polyphenols (TP) as well as individual ones (UPLC ESI MS^2^) and antioxidant capacity (FRAP, DPPH and ORAC). Moreover, the biological activity of the selected extracts was additionally determined. The optimal PLE and MAE conditions were 150 °C, 5 min extraction time and SSR 1:30 g/mL (TP 80.0 mg GAE/g dm), and 70 °C, extraction time 5 min and SSR 1:30 g/mL (TP 36.4 mg GAE/g dm), respectively. Both methods yielded similar polyphenol profiles (43 compounds) but differed quantitatively. MAE extracts contained more flavonols and phenolic acids, while PLE extracts had higher procyanidins and flavan-3-ols. Furthermore, the PLE extract exhibited a superior antioxidant capacity. This BCL extract also showed that it can protect against oxidative and DNA damage and can induce free radical formation and DNA damage, albeit at different doses. Moreover, it had a moderate antimicrobial activity against *S. aureus* and *B. subtilis*, while no antimicrobial activity was observed against Gram-negative bacteria as well as yeasts, lactic acid bacteria and molds.

## 1. Introduction

Over the last decade, a circular economy model has been developed, based on principles that seek to overcome the current production and consumption model, which is characterized by increasing consumption and depletion of resources [1]. As the circular economy promotes sharing, renting, reusing, repairing, renovating and recycling existing materials and products for as long as possible [2] and reducing waste to a minimum [3], various efforts are being made to find ways to reuse available resources and, naturally, with the aim of reducing waste. In this way, materials that are often considered undesirable and unusable can be given a new chance to be used, often even with added value.

Black chokeberry (*Aronia melanocarpa* L.) leaves (BCL) that remain after the fruit has been harvested are usually regarded as waste, although they have great potential for further utilization due to their high content of bioactive compounds—dominantly polyphenols [4,5,6,7]. Previous studies have confirmed the presence of hydroxybenzoic and hydroxycinnamic acids, flavanols, flavonols and even stilbenes in small amounts, with chlorogenic acid and epicatechin being the major constituents according to Saracila et al. [7]. Other authors have also reported a similar profile of polyphenols in BCL, although quercetin derivatives were the major polyphenols, along with chlorogenic acid and its isomers [4,5,6]. The high presence of these natural antioxidants enables various biological activities of BCL extract such as antioxidant, hypoglycemic, antineurodegenerative, antidiabetic, anti-tyrosinase, anti-inflammatory, antitumor [8,9,10,11,12], antimicrobial [12,13] and others. Therefore, precisely because of the high content of polyphenols and beneficial biological effects, BCL can be considered a valuable raw material for the extraction of these bioactives, which can then be incorporated into various matrices and thus contribute to the functionality of the product in the food, cosmetics and pharmaceutical sectors. From this perspective, the BCL are given a new value because they have great potential for reuse instead of being discarded after harvesting, which is in line with the principles of the circular economy and sustainability.

However, it is important to realize that in order to fully exploit the potential of BCL in terms of isolating their polyphenols, it is crucial to carefully select the extraction method and its parameters to maximize the efficiency in obtaining the target compounds. In the field of extraction, there are various methods depending on the raw material or target compounds, both conventional ones such as Soxhlet, reflux, maceration, percolation, etc., and advanced ones such as pressurized liquid extraction (PLE), microwave-assisted extraction (MAE), ultrasound-assisted extraction and many others. The latter have attracted a great interest from scientists in the last decade as they represent a “green” approach for the isolation of various compounds, as they are characterized by high productivity alongside lower solvent consumption and lower extraction time and thus energy, which has a positive impact on the environment. Precisely because of these features, these advanced methods have an advantage over conventional extraction methods. PLE and MAE have previously demonstrated their high efficiency in extracting various bioactive compounds from plant material [14,15,16]. Although their principles differ, both techniques show a more than satisfactory extraction outcome in a reasonable extraction time. The PLE principle manifests itself in the application of high pressure and high temperature, which allows the solvent to be kept above its boiling point in a liquid state, which directly enables better solubility and extraction of the target compounds. As the extraction is carried out in a closed system, the degradation of oxygen-sensitive compounds is also minimized [17]. The MAE principle, on the other hand, involves microwave heating caused by ion conduction and dipole rotation, which leads to a rupture of the cell walls and better penetration of the solvent into the matrix, enabling effective extraction of the target compounds [18]. However, it should be remembered that for maximum efficiency, the extraction parameters for both PLE and MAE must be meticulously and thoroughly selected in conjunction with the matrix type and the nature of the target compounds. In PLE, the most important parameters to be set are the extraction temperature, the static extraction time and the number of extraction cycles, while the pressure is usually kept at a high level (e.g., 10.34 MPa). In contrast, temperature, irradiation time and microwave power are the most commonly required MAE parameters to be specified. The general rule that the diffusion rate and solubility of the compounds of interest increases with increased temperature and longer extraction time therefore applies to both techniques, but care should be taken with regard to the possible degradation of heat-sensitive compounds. In addition, the increase in microwave power during MAE leads to the increase in extraction temperature due to increased molecular interactions in the electromagnetic field, which has a direct effect on the extraction outcome [19,20].

Furthermore, sample preparation in terms of solvent type and sample-to-solvent ratio (SSR) also plays an important role in maximizing extraction performance to ensure the complete extraction of target compounds. Previous studies have confirmed that aqueous-alcoholic mixtures are the best performing in polyphenol extraction, with ethanol being mostly preferred as it is GRAS and affordable. As some polyphenols are more water-soluble, the presence of water in the ethanolic mixture allows their release, which is why aqueous mixtures with ethanol are mostly used [18,21,22]. As for SSR, increasing the amount of solvent leads to an increase in the concentration gradient, which increases the diffusion rate and improves extraction efficiency. However, large amounts of solvent are associated with additional costs. On the other hand, when a small amount of solvent is used, saturation occurs and extraction efficiency decreases [22]. Therefore, it is highly desirable to determine a suitable amount of solvent to achieve maximum extraction efficiency while maintaining cost efficiency.

Although the available literature provides an insight into different extraction methods of polyphenols from BCL, to the best of the author’s knowledge, there is no study combining and comparing PLE and MAE. Furthermore, no data on PLE from BCL were found. Therefore, the aim of this study was to determine the optimal PLE and MAE conditions, i.e., extraction temperature, time and SSR, to obtain the maximum yield of polyphenols from BCL and to compare the efficiency of these extraction techniques. Additionally, this study also aimed to determine the biological and antimicrobial effects of the extract from BCL obtained under the optimal conditions of the selected extraction technique.

## 2. Materials and Methods

### 2.1. Chemicals and Reagents

Distilled water was produced using the Milli-Q water purification system (Millipore, Bedford, MA, USA). Ethanol (96%), iron (III) chloride hexahydrate and methanol were purchased from Gram-mol (Zagreb, Croatia). 2,4,6-tri(2-pyridyl)-s-triazine (TPTZ), 6-hydroxy-2,5,7,8-tetramethylchroman-2-carboxylic acid (Trolox), 2,2-diphenyl-1-(2,4,6-trinitrophenyl)hydrazyl (DPPH), 2,2′-Azobis(2-amidinopropane) dihydrochloride (AAPH), 3-(4,5-dimethylthiazol-2-yl)-2,5-diphenyltetrazolium bromide (MTT), neutral red dye, 2′− 7′- dichlorodihydrofluorescein diacetate (DCFH-DA), disodium ethylenediaminetetraace-tate dihydrate (Na_2_EDTA), tris(hydroxymethyl)aminomethane (Tris base) and Triton X-100, low melting point agarose (LMP), normal melting point agarose (NMP) and resazurin sodium salt were obtained from Sigma-Aldrich (St. Louis, MO, USA). Hydrochloric acid (37%) and glacial acetic acid were purchased from Carlo Erba Reagents (Val-de-Reuil, France); Folin-Ciocolateu, sodium dodecyl sulfate (SDS), crystal violet, ethidium bromide and Giemsa stain from Merck (Darmstadt, Germany), sodium carbonate, sodium hydroxide, sodium chloride and dipotassium phosphate from Kemika (Zagreb, Croatia); and acetonitrile and fluorescein from Honeywell Riedel-de-Haën (Seelze, Germany). Formic acid was procured from BDH Prolabo (Lutterworth, UK). Mueller–Hinton agar and broth, De Man–Rogosa–Sharpe (MRS) agar and Mueller–Hinton agar with 2% glucose were obtained by Merck Millipore (Darmstadt, Germany). Antimicrobial susceptibility discs (kanamycin and nystatin) were purchased from BioLab Inc. (Budapest, Hungary). Working standards of apigenin, epigallocatechingallate, luteolin, catechin and epicatechingallate were purchased from Extrasynthese (Genay, France), while *p*-coumaric acid, caffeic acid, ferulic acid, procyanidin B2, rutin, kaempferol-3-rutinoside, myricetin, quercetin-3-glucoside, luteolin-6-C-glucoside, gallic acid, rosmarinic acid, chlorogenic acid and protocatechuic acid were obtained by Sigma-Aldrich. Cell culture media (Ham’s F-12 nutrient mixture) and medium constituents (fetal bovine serum, FBS) were purchased from Capricorn (Düsseldorf, Germany).

### 2.2. Plant Material

BCL (*A. melanocarpa* L.) harvested in August 2023 from the orchard of Terra Food Ltd. (Koprivnica, Croatia) were used in this study. After harvesting, the samples were air-dried until there was appropriate dry matter (90.15%). Immediately before extraction, the dry BCL samples were ground (particle size 0.45–1 mm) using an electric grinder.

### 2.3. PLE

The PLE of polyphenols from dried BCL was performed by the Thermo Scientific^TM^ Dionex ASE^TM^ 350 Accelerated Solvent Extractor (Sunnyvale, CA, USA). Extractions were carried out in 34 mL stainless steel cells equipped with two cellulose filters containing a certain amount of sample according to the experimental design mixed with diatomaceous earth. An aqueous solution of ethanol (30%, *v*/*v*) was used as solvent. To determine optimal conditions for achieving maximum extraction efficiency, extractions were performed varying the extraction temperature (100, 125 and 150 °C), static extraction time (5 and 10 min) and SSR (1:20, 1:30, 1:40 g/mL), as shown in Table 1. Constant parameters were pressure of 10.34 MPa, 3 extraction cycles, 30 s of nitrogen purge and 30% of volume flush. After completion of the extraction, the extracts were filtered into 50 mL volumetric flasks and filled up to the mark with a solvent. The extracts prepared were stored in plastic Falcon tubes at +4 °C until analyzed.

### 2.4. MAE

Polyphenols were extracted from dried BCL using the Ethos Easy Reactor (Milestone, Sorisole, Italy). The process involved weighing the appropriate amount of crushed sample into the extraction cell based on the specified SSR, then adding 40 mL of extraction solvent (30% ethanol) and a magnetic stirrer. The cell containing the mixture was placed into the reactor, and extraction was performed following the experimental design (Table 1), where the parameters varied were temperature (60, 70 and 80 °C), extraction time (5 and 10 min) and SSR (1:20, 1:30, 1:40 g/mL). Constant parameters were a microwave power of 400 W, 3 min preheating time, 1 min for ventilation and cooling post-extraction and a stirring intensity set at 50%. After extraction, the extracts were filtered into 50 mL volumetric flasks and made up with a solvent. The extracts prepared were stored in plastic Falcon tubes at +4 °C until analyzed.

### 2.5. Determination of Total Polyphenols

Total polyphenols (TP) were determined according to the modified method of Shortle et al. [23]. Briefly, 100 μL of the extract, appropriately diluted with extraction solvent, 200 μL of the Folin-Ciocalteu reagent, 2 mL of distilled water and, after 3 min, 1 mL of sodium carbonate solution (20%, *w*/*v*) were pipetted into the test tube. The blank sample was prepared in the same way as the sample, but instead of the extract, 100 μL of the extraction solvent was taken. The prepared samples were mixed in a vortex mixer and thermostated in a water bath at 50 °C for 25 min. The absorbance was then measured at 765 nm. A calibration curve was established using gallic acid standard solutions ranging from 50 to 500 mg/L. Results are expressed in mg of gallic acid equivalents (GAE)/g of dry matter (dm).

### 2.6. Determination of Individual Polyphenols

The individual polyphenols’ composition of the optimized PLE and MAE extract of BCL was determined by UPLC ESI MS^2^ analysis on an Agilent 1290 series RRLC instrument (Agilent, Santa Clara, CA, USA) with a 6430 triple quadrupole mass spectrometer featuring an ESI ion source. The ESI ionization was conducted in both positive and negative modes (*m*/*z* 100 to 1000), with nitrogen (99.999%, Messer, Zagreb, Croatia) as the induction cone and collision gas under positive/negative capillary voltage 4000/3500 V, drying gas temperature 300 °C, flow rate 11 L/h and nebulizer pressure 40 psi. Chromatographic separations took place on a Zorbax Eclipse Plus C18 column (Agilent, 100 × 2.1 mm; 1.8 µm particle size) at 35 °C, with an injection volume of 2.5 µL. Data collection and processing were conducted with the Agilent MassHunter workstation software (ver. B.04.01). The method, solvent composition, and quality parameters, including calibration curves, instrumental limits of detection (LOD) and quantification (LOQ), were previously described by Elez Garofulić et al. [24]. Identification and quantification were based on calibration curves for the following standards: gallic, chlorogenic, rosmarinic, caffeic, protocatechuic, ferulic acids, quercetin-3-glucoside, quercetin-3-rutinoside, kaempferol-3-rutinoside, myricetin, catechin, epigallocatechin gallate, epicatechin gallate, luteolin, apigenin and procyanidin B2. For compounds without commercial reference standards, tentative identification was based on mass spectral data and literature reports of mass fragmentation patterns. Quantification involved using chlorogenic acid calibration for quinic, 4,5-dicaffeoylquinic and 3-*p*-caffeoylquinic acids, gallic acid calibration for *p*-hydroxybenzoic acid, ferulic acid calibration for 3-*O*-ferulloylquinic acid, quercetin-3-glucoside calibration for quercetin, quercetin dihexoside, rhamnoside, vicianoside, pentosylhexoside, glucuronide and pentoside as well as for isorhamnetin glycosides, the kaempferol-3-rutinoside calibration curve for all identified kaempferol glycosides, myricetin for all myricetin glycosides, the apigenin calibration curve for its pentoside and luteolin for its rutinoside, the catechin calibration curve for epicatechin and the procyanidin B2 curve for the calculation of procyanidin B1 and procyanidin trimer. All results are expressed in mg/g dm.

### 2.7. Determination of Antioxidant Capacity

#### 2.7.1. Ferric Reducing Antioxidant Power (FRAP) Analysis

The FRAP assay was conducted following the method outlined by Benzie and Strain [25], with several modifications. Firstly, 240 μL of distilled water, 80 μL of sample and 2080 μL of FRAP reagent were pipetted into a glass test tube. For the blank, 80 μL of extraction solvent was used instead of the sample. The prepared mixtures were then vortex-mixed and incubated at 37 °C for 5 min, after which the absorbance was measured at 593 nm. The results are reported as µmol of Trolox equivalents (TE)/g dm based on the Trolox standard calibration curve.

#### 2.7.2. 2,2-Diphenyl-1-picrylhydrazyl Radical (DPPH) Scavenging Analysis

The determination of the antioxidant capacity using the DPPH method was carried out according to the method of Braca et al. [26]. In short, 0.75 mL of the extract and 1.5 mL of a 0.2 mmol/L DPPH solution were mixed in a glass tube. The blank sample contained 2.25 mL of 100% methanol. The prepared samples stood for 20 min at room temperature in the dark, and afterwards the absorbance was measured at 517 nm. The results are reported as µmol TE/g dm based on the Trolox standard calibration curve.

#### 2.7.3. Oxygen Radical Absorbance Capacity (ORAC) Analysis

The ORAC assay was performed on a 96-well microplate using a fluorescence plate reader (Clariostar, BMG LABTECH, Offenburg, Germany) according to the method described by Elez Garofulić et al. [27]. The sodium phosphate buffer (75 mM, pH 7.4) was used as a blank (25 μL) and Trolox (25 μL) as a standard. Data were analyzed by MARS 2.0 software. The results are expressed as µmol TE/g dm.

### 2.8. Determination of Biological Activity

The biological activity of the selected BCL extract was evaluated on a tongue epithelial carcinoma cell line (CAL 27; ATCC CRL-2095) and a human hepatocellular carcinoma cell line (HepG2; ATCC HB-8065), where its effects on toxicity, survival and clonogenic (colony-forming) growth were examined. Additionally, the antioxidant properties of the extract in cells and its influence on the genetic material of the cells were also investigated.

For the analyses of biological activity, selected BCL extract was evaporated (Vacuum concentrator SpeedVac Concentrator SPD2010-230, Thermo Fisher Scientific, Sunnyvale, CA, USA) under 50 °C, vacuum pressure 8 and vacuum ramp 5. The extract was firstly evaporated to dryness, then frozen at −80 °C for 24 h, and subsequently freeze-dried (Alpha 1–4 LSCplus freeze-drying system, Martin Christ Gefriertrocknungsanlagen GmbH, Osterode am Harz, Germany) for 48 h at −55 °C.

The resulting freeze-dried extract was then used to prepare initial solutions containing 5 mg/mL of polyphenols. These initial solutions were further diluted to create working samples with polyphenol concentrations of 0.014, 0.07, 0.2 and 0.5 mg/mL. The selected range of concentrations was based on the recommended daily intake of polyphenols. CAL 27 cells were treated with the indicated extract concentrations for 30 min, while HepG2 cells were treated for 4 h. All experiments were performed in at least 4 replicates, and the experiment was repeated three times. All absorbance and fluorescence measurements in multiwell plates were performed using a FLUOstar OPTIMA plate reader (BMG Labtech, Durham, NC, USA).

#### 2.8.1. Cell Lines

Human cell lines were cultured in Ham’s F-12 medium supplemented with L-glutamine and 10% fetal bovine serum (FBS), and maintained at 37 °C in a humidified 5% CO_2_ atmosphere. For performing neutral red, MTT and DCFH-DA assays, cells were seeded in 96-well plates at a concentration of 105 per well. The following day, cells were treated with 4 different concentrations of extract prepared in growth medium. The cell lines utilized in this investigation were graciously supplied by the Ruđer Bošković Institute, Zagreb, Croatia (CAL 27), and the Institute for Medical Research and Occupational Health, Zagreb, Croatia (HepG2).

#### 2.8.2. Toxicity and Cell Survival

##### Neutral Red Assay

Cytotoxicity was determined using the neutral red (NR) assay [28]. Following treatment, the testing solutions were removed, and the cells were washed twice with 100 μL of PBS saline buffer. Then, a medium containing 40 μg/mL of NR dye was added to the cells and incubated for 45 min to allow the dye to be absorbed. After discarding the NR solution, the absorbed dye was extracted from the cells using a destaining solution composed of 50% ethanol, 49% distilled water and 1% glacial acetic acid. Absorbance was measured at 540 nm. The percentage of surviving cells was calculated in comparison to the 100% negative control.

##### MTT Assay

The MTT assay is a colorimetric assay for assessing cell metabolic activity. NAD(P)H-dependent cellular oxidoreductase enzymes may, under defined conditions, reflect the number of viable cells present [29]. Following the treatment, 100 μL of MTT reagent (0.5 M) was added to each well. After a 4-h incubation period, the MTT reagent was replaced with 100 μL of 10% SDS, and the cells were then incubated overnight at room temperature in the dark. The absorbance was measured at λ = 595 nm. The results are presented as the percentage of surviving cells compared to the 100% control values.

##### Clonogenic Assay

The clonogenic assay, also known as the colony formation assay, is an in vitro method for evaluating cell survival. This technique relies on the ability of individual cells to proliferate and form colonies [30]. The 50 CAL 27 and HepG2 cells were seeded into individual wells of a 24-well cell culture plate, culturing them in growth medium supplemented with 10% FBS. After a 24-h incubation, the cells were treated with a previously defined range of extract concentrations for 30 min and 2 h. Following 7 days of cultivation in a fresh growth medium, visible colonies appeared in the wells, and these cells were then stained with crystal violet (0.01%, *w/v*) and Giemsa (0.1%, *w/v*). Plating efficiency (PE) and survival fraction (SF) were calculated based on the number of counted colonies for the control and each sample.

#### 2.8.3. Evaluation of Antioxidative and DNA Damage Protective Effect

##### DCFH-DA Assay

The formation of reactive oxygen species (ROS) in the studied cell lines was determined using a 2′,7′-dichlorofluorescein-diacetate (DCFH-DA) fluorometric assay [31]. Following incubation with the tested extracts, the cells were washed with PBS and exposed to a 100 μL DCFH-DA solution for 30 min. The fluorescence intensity was then measured at an excitation wavelength of 485 nm and an emission wavelength of 520 nm. The results are expressed as the relative increase or decrease in fluorescence intensity.

##### Comet Assay

The comet assay is a commonly used method for the assessment of oxidative DNA damage and corresponding repair mechanisms [32]. In 24-well plates, the cells were treated and seeded at a density of 105 cells/mL according to the prior description. The cells were washed and scraped, and a single-cell suspension was then centrifuged at 1000× *g* for 5 min at 4 °C. A total of 100 µL of 0.5% low melting point agarose (LMP) in phosphate-buffered saline (PBS) was mixed with the resultant pellet. After pre-coating a dried microscope slide (Surgipath, Richmond, IL, USA) with 300 µL of 1.5% normal melting point agarose (NMP, Sigma-Aldrich, USA) in PBS buffer, the agarose-cell combination was applied. A 100 µL layer of 0.5% agarose gel was added after the cell layer had solidified, and it was covered with a coverslip. The slides were submerged for 1 h at 4 °C in a lysis solution containing 100 mM Na_2_EDTA, 2.5 M NaCl and 10 mM Tris (pH = 10) enhanced with 1% Triton-X after the gels were left to solidify on ice for 10 min. The coverslips were then removed. The slides were first cleaned with distilled water, and then they were electrophoresed for 20 min at 25 V in a cold, freshly made denaturation and electrophoresis buffer (10 mM NaOH), 200 mM Na_2_EDTA, pH 13). After three rounds of neutralization, each lasting 5 min with 0.4 M Tris/HCl, pH 7.5, the slides were stained with 50 µL of ethidium bromide solution (20 μg/mL) per slide for 10 min. The results were assessed using an image analysis system (Comet assay IV, Perceptive instruments Ltd., Instem, UK) and a fluorescence microscope (Olympus BX51×, Tokyo, Japan, with a 200× magnification). Only comets with a clearly defined head were scored in the measurements of 100 randomly chosen cell images. As trustworthy markers of genotoxicity, three parameters—tail length (presented in µm), tail intensity (i.e., DNA% in tail) and tail moment (tail length × % of DNA in the tail)—were utilized to evaluate DNA damage [33]. Before conducting the data analysis, a natural logarithm (ln) transformation was applied to normalize the data distribution and equalize the variances [34].

##### Plasmid ΦX174 RF1 DNA

Agarose gel electrophoresis was used to quantify DNA cleavage by ROS generated through the use of H_2_O_2_ and UV, following the modified protocol outlined by Keum et al. [35]. Briefly, the reaction mixture (20 µL) contained plasmid ΦX174 RF1 DNA (0.3 μg, Promega Corporation, Madison, WI, USA), TE buffer (1 mM EDTA and 10 mM Tris-HCl, pH 8.0), H_2_O_2_ (0.03 M) and a final concentration of the extract. The reaction mixtures were exposed to UV irradiation for 7 min at a distance of 50 cm using a 30 W germicidal UV lamp to produce hydroxyl radicals. After a 30-min incubation at room temperature, the reaction was terminated by adding a 6× DNA loading buffer which contained 30% glycerol (*v/v*) and 0.25% bromophenol blue dye (*w/v*). The mixtures were analyzed via 1% agarose gel electrophoresis (60 V, 2 h) in TAE buffer. The gels were stained with ethidium bromide (0.5 μg/mL), then distained in water before being imaged using a UV transilluminator. The results are reported as the percentage of preserved supercoiled DNA relative to the negative control (100%).

### 2.9. Determination of Antimicrobial and Antifungal Activity

#### 2.9.1. Agar Well Diffusion Antimicrobial Assay

The antimicrobial activity of the selected BCL extract was evaluated against Gram-positive bacteria (*Staphylococcus aureus*, *Bacillus subtilis* and *Enterococcus faecium*), Gram-negative bacteria (*Pseudomonas aeruginosa*, *Escherichia coli* and *Salmonella enterica s.* Typhimurium), lactic acid bacteria (*Lactobacillus brevis*), yeasts (*Candida albicans*, *Candida utilis* and *Saccharomyces cerevisiae*) and mold *Penicillium* sp. using a well diffusion method. Prior to analysis, the BCL extract was evaporated to dryness as described in Section 2.8. and appropriately dissolved in sterilized distilled water to obtain 19.1 mg GAE of TP/mL. After overnight growth of the cultures under anaerobic conditions in the appropriate culture media, microbial suspensions were prepared by dilution with PBS, and cell density was adjusted to ≈106 cells/mL. Sterile Petri dishes, each containing 20–25 mL of the Mueller–Hinton agar (Gram-positive and Gram-negative bacteria), MRS agar (lactic acid bacteria) and Mueller–Hinton agar with 2% glucose (yeast and mold), were inoculated with a sterile cotton swab from the microbial suspension according to the Clinical and Laboratory Standards Institute (CLSI) protocol. Three 8 mm diameter wells were then made on the solidified surface of the medium on each dish and a disc of kanamycin (50 µg) or nystatin (100 U) was added as a positive control. Subsequently, 0.1 mL of BCL extract (aqueous extract with TP of 19.11 mg GAE/mL) was added to each wall (triplicates of sample). The Petri dishes were then incubated for 18–24 h at 28–37 °C for the yeast, mold or bacteria. After the incubation period, the growth inhibition zones were measured and the results are expressed as the arithmetic mean of three measurements. The antimicrobial activity was expressed on the basis of the diameter of the growth inhibition zone and compared to the positive control kanamycin or nystatin as follows: <10 mm—no antimicrobial activity; 10–15 mm—weak antimicrobial activity; 16–20 mm—moderate antimicrobial activity; >20 mm—certain antimicrobial activity.

#### 2.9.2. Determination of Minimum Inhibitory Concentration and the Number of Living Cells

The minimum inhibitory concentration (MIC) was determined with the resazurin cell viability assay using the broth microdilution method [36]. In brief, Mueller–Hinton broth was used for bacterial growth. A fresh 24-h broth culture was used (*S. aureus* and *B. subtilis*), and the final density was adjusted to 1 × 10^7^ colony-forming units (CFU)/mL. Two-fold dilution series of the test extract were prepared in 200 μL of the medium. The extract was tested in a concentration gradient of 9.55 mg of polyphenols/mL–0.30 mg polyphenols/mL. During the experiment, a growth control was maintained without the extract (negative control) and with kanamycin (positive control). The microtiter plate was incubated at 37 °C for 24 h, after which 0.03% resazurin dye was added (50 µL). The plate was further incubated for 2 h to observe the color change. The blue dye is converted to pink in the presence of metabolically active cells and this conversion can be detected by visual observation. The lowest concentration of the extract at which no color change of the resazurin occurred was determined as the MIC. Three replicates were performed for each bacterium. The number of live cells was determined both in the MIC wells and in the wells without extracts (negative control). To determine the number of live cells, serial 10-fold dilutions were prepared in sterile saline and aseptically plated on Mueller–Hinton agar plates. All agar plates were incubated at 37 °C for 24 h and the colonies were counted to determine the CFU/mL.

### 2.10. Experimental Design and Statistical Analysis

For the experimental design and statistical analysis Statistica ver. 12.0 (Statsoft Inc., Tulsa, OK, USA) software was used. To evaluate the influence of PLE and MAE conditions, a mixed 2- and 3-level full factorial experimental design was applied, comprising a total of 18 trials for each extraction technique. The independent factors were temperature, static extraction time and SSR for PLE and temperature, and time and SSR for MAE, respectively. The dependent variable was the content TP of the BCL for both extraction techniques. All extractions and analyzes were carried out in duplicate, except analyzes of biological activity which were performed in at least 4 replicates, the experiment was repeated three times, and antimicrobial activity was carried out in triplicate. The results are expressed as mean ± standard deviation (SD), while the results of the statistical analysis are expressed as mean ± standard error (SE). The residuals of the data were tested for normality and homoscedasticity using the Shapiro–Wilk test and Levene test, respectively. The data were further analyzed by analysis of variance (ANOVA) followed by a post-hoc Tukey’s HSD test (parametric data) or by the Kruskal–Wallis test in conjunction with a multiple comparison of mean ranks (non-parametric data), where appropriate. For the comparison of individual polyphenols and antioxidant capacity values between PLE and MAE extracts obtained under optimal extraction conditions, and biological activity of the selected BCL extract, one-way ANOVA coupled with post-hoc Tukey’s HSD test was used. A significance level of *p* ≤ 0.05 was set for all tests.

## 3. Results and Discussion

### 3.1. Optimization of PLE and MAE

The results for TP determined in the BCL extracts obtained under different extraction conditions of PLE and MAE are given in Table 1. In PLE extracts, the TP varied between 29.0 and 80.0 mg/g dm, and while using MAE it ranged from 23.9 to 49.9 mg/g dm. These results are in agreement with the results of the study on leaves of different chokeberries [6], but also lower than those reported by Zdunić et al. [8] who used maceration as an extraction technique with 70% ethanol as solvent during 14-*h* of extraction at room temperature. Similarly, Do Thi and Hwang [37], achieved higher values by using maceration for 2 h with either distilled water at 100 °C or 80% ethanol at 85 °C. In general, the difference in TP could be due to the choice of a different chokeberry variety and the extraction technique, but also the solvent, temperature and the time of extraction. In this study slightly higher values were generally obtained with PLE, which may be a consequence of the application of high temperature and pressure, which increased the solubility of the sample due to a better diffusion rate and mass transfer, as well as performing the extraction in several cycles, thus supplying fresh solvent and allowing maximum extraction of the target components.

The influence of the PLE and MAE parameters (temperature, time and SSR) on the TP of the BCL extracts is shown in Table 2. As can be seen, only temperature had a statistically significant influence on the TP in extracts obtained by PLE, while time and SSR were not statistically significant. The proportion of polyphenols increased with increasing temperature. This was due to a better penetration of the solvent into the pores of the matrix at elevated temperatures, as the solvent remains in a liquid state above the boiling point under the influence of high pressure during PLE [21], and the solubility and diffusion of the target molecules into the solvent was improved. Other studies also concluded that an increase in temperature in PLE leads to an increase in the TP on leaves of blackcurrant [15] and black bamboo [38]. Although the SSR did not significantly affect the TP during PLE, some differences can be observed. When raising the SSR from 1:20 to 1:30 g/mL, there was a slight increase in the amount of TP in the BCL extracts, while a further increase in the solvent volume did not cause an additional enhancement in TP, suggesting that extraction equilibrium was achieved. Generally, an increased solvent volume improves the extraction efficiency as it enhances the solubility of target compounds as well as the mass transfer between the solvent and plant material. However, this occurrence manifests up to the point where the extraction equilibrium is reached. Elez Garofulić et al. [15] came to the same conclusion while investigating the influence of PLE parameters on polyphenols from blackcurrant leaves. Nevertheless, according to the findings, the optimal parameters for the efficient isolation of the TP from the BCL by PLE were a temperature of 150 °C, 5 min extraction time and 1:30 g/mL SSR, whereby the TP was obtained in an amount of 80.0 mg GAE/g dm.

In the case of MAE, temperature and SSR had a statistically significant influence on the TP in the BCL extracts, whereas time did not (Table 2). Increasing the temperature from 60 to 70 °C resulted in a significant increase in TP, after which the TP remained constant. At higher temperatures, polyphenols may be less stable and more susceptible to thermal degradation, leading to a decrease in their content. This conclusion was also reached by Xie et al. [39] who isolated flavonoids from *Cyclocarya paliurus* (Batal.) Iljinskaja leaves by MAE. With an increase in SSR from 1:20 to 1:30 g/mL there was an increase in TP, while a further increase to 1:40 g/mL showed no statistical difference in the TP. Dahmoue et al. [40] investigated SSR between 1:20 and 1:40 g/mL for the MAE of polyphenols from *Myrtus communis* L. leaves and concluded that the optimal SSR was 1:32 g/mL. Furthermore, Eskilsson and Bjorklund [41] stated that in MAE, smaller amounts of solvent may be sufficient for the extraction of polyphenols, since using too much solvent makes it hard for microwaves to penetrate deep inside as the energy is absorbed at the surface. Therefore, it is important to optimize the extraction process for each plant species analyzed. Based on the above, 70 °C, an extraction time of 5 min and SSR of 1:30 g/mL, yielding 36.4 mg GAE/g dm of TP, can be considered optimal MAE parameters for effective extraction of TP from BCL.

### 3.2. Polyphenolic Characterization of BCL Extracts Obtained Under Optimal PLE and MAE Conditions

BCL extracts obtained under optimal PLE and MAE conditions were further analyzed by UPLC ESI MS^2^ for their chemical profile in terms of individual polyphenols. Detailed composition of polyphenols in these extracts is given in Table 3.

A total of 43 polyphenols were identified in PLE and MAE extracts of BCL, including 9 phenolic acids, 23 flavonols, 4 flavones, 4 flavan-3-ols and 3 procyanidins. The compounds lacking authentic commercial standards were identified according to their characteristic fragmentation pathways. The flavonols were determined to be the predominant phenolic class in the BCL, mainly represented by quercetin, kaempferol and their respective glycosides resulting in a total concentration of 24.25 to 27.43 mg/g dm. This concentration range is in line with one reported by Lee et al. [5], but lower than that reported by Staszowska-Karkut and Materska [42] and Saracila et al. [7]. Kaempferol aglycone was the major flavanol, followed by quercetin and quercetin rutinoside. A significantly lower proportion of the total identified polyphenols was assigned to phenolic acids, which were mainly represented by chlorogenic acid. The predominance of chlorogenic acid in BCL has been confirmed by other authors [9]. However, Lee et al. [5] reported that caffeoylquinic acid derivatives (including chlorogenic acid) accounted for more than 70% of the TP of young BCL, with their content decreasing significantly over the maturation period resulting in less than 6% in aged leaves. Thus, the maturity stage of the leaves is the major factor in determining the final composition of polyphenols in BCL. Apart from flavanols, the flavonoids in BCL were characterized by flavan-3-ols and procyanidins and, to a lesser extent, by flavones. Luteolin was found in the highest concentration among the flavones, while epicatechin and procyanidin B1 were the dominant compounds from the class of flavan-3-ols and procyanidins, respectively.

Saracila et al. [7] found epicatechin to be the major flavonoid in BCL at a concentration of 4.25 mg/g dw and Owczarek et al. [43] reported the presence of procyanidin B2, while other flavan-3-ols and procyanidins identified in this study were not previously reported. The flavones, which were determined at low concentrations, were reported in only one study [5].

The application of two different extraction techniques, based on different mechanisms, did not affect the qualitative composition of the BCL extracts; however, it caused some differences in their quantitative profile. The MAE resulted in an extract with a higher content of two major phenolic classes, namely flavonols and phenolic acids, while the PLE only favored the yield of procyanidins and flavan-3-ols. The outperformance of MAE in isolating phenolic acids could be explained by the general observation that compounds with fewer substituents in the aromatic ring are more stable under microwave irradiation [44], while flavonols, although having a more complex structure, might prefer the moderate temperature conditions of MAE in comparison to the significantly higher ones applied in PLE. However, flavan-3-ols and procyanidins showed a different behavior as they obtained a higher extraction yield under pressurized conditions and at higher temperatures. This could be related to the behavior of compounds with a complex structure and more hydroxyl substituents, which under pressurized conditions increase the number of hydrogen bonds with the solvent as a consequence of reduced tension between the solvent and the material, thus promoting their solubility [45,46]. One can also notice an opposite trend in the TP determined spectrophotometrically and by UPLC ESI MS^2^ between the PLE and MAE extract. While the TP determined spectrophotometrically was higher in the PLE extract, the amount determined by UPLC ESI MS^2^ was higher in the MAE extract. This occurrence may be related to the different selectivity of these extraction techniques, with PLE having a lower selectivity than MAE and generally extracting more other compounds, such as pigments, in addition to polyphenols. The same phenomenon was previously also reported by Elez Garofulić et al. [47]. Moreover, since the Folin–Ciocalteu reagent reacts not only with polyphenols, but also with other compounds, such as sugars, organic acids and chlorophylls, the amount of TP determined spectrophotometrically could be associated with those compounds and therefore increased [48,49,50].

### 3.3. Antioxidant Capacity of BCL Extracts Obtained Under Optimal PLE and MAE Conditions

To evaluate the antioxidant properties of the BCL extracts obtained under optimal PLE and MAE conditions, their antioxidant capacity was determined using the FRAP, DPPH and ORAC methods. The results obtained show a high antioxidant capacity of both BCL extracts, with the PLE extract having the highest value in the DPPH assay (802.2 µmol TE/g dm) (Table 4). The same extract had a FRAP value of 659.4 µmol TE/g dm, while the ORAC value was the lowest determined (578.9 µmol TE/g dm). 

The antioxidant capacity values of the MAE extract followed the same descending order with respect to the assay used, i.e., DPPH 531.7 µmol TE/g dm, FRAP 489.6 µmol TE/g dm and ORAC 479.1 µmol TE/g dm. A previous study [7] confirmed the highest antioxidant capacity of BCL measured by different methods, including DPPH, compared to fruit and pomace, while Lee et al. [5] confirmed that the antioxidant capacity of BCL decreases with their maturity. The FRAP results obtained in this study are in general agreement with the results of Szopa et al. [6] who also evaluated the antioxidant capacity of BCL. They also performed a DPPH analysis, but the results are difficult to compare due to the different expression of the results. Zdunić et al. [8] also confirmed the high antioxidant capacity of *A. melanocarpa* leaves and obtained the highest FRAP value (893.73 μmol FeSO4·7H_2_O/g dry extract) in the *n*-butanol fraction (500 μg/mL) obtained after maceration of the leaves with 70% ethanol. The same fraction showed 87.02% of DPPH radical scavenging activity.

With regard to the extraction technique, it can be clearly seen that the PLE extract outpowered the MAE extract in terms of antioxidant capacity, achieving significantly higher values with all three methods (Table 4). The different values of antioxidant capacity of the PLE and MAE extracts are consistent with the amounts of TP determined spectrophotometrically in these extracts, which leads to the conclusion that the antioxidant capacity of these extracts derives not only from polyphenols but also other bioactive compounds present, confirming the previous statement about the different selectivity of these extraction techniques. Higher levels of antioxidant capacity of blackcurrant and bilberry leaves in PLE extracts in comparison with their MAE extracts have also been previously reported by Elez Garofulić et al. [15]. They used the same assays as in this study, including an additional one (2,2-azinobis(3-ethylbenzothiazoline-6-sulfonic acid, ABTS), and the results for FRAP, ABTS and ORAC in the blackcurrant leaves extract were higher when using PLE. Only the DPPH value was higher in the MAE extract of blackcurrant leaves. Furthermore, in their study, the bilberry extract showed a higher value of antioxidant capacity determined by FRAP, ABTS and DPPH when PLE was applied, while ORAC values were almost equal in both extracts of PLE and MAE.

As the PLE extract of BCL showed a stronger antioxidant capacity and apparently contained other bioactive compounds besides polyphenols, it was selected for further analysis of its biological activity.

### 3.4. Toxicity of BCL Extract Obtained Under Optimal PLE Conditions and Cell Survival After Treatment

**Figure 1:**
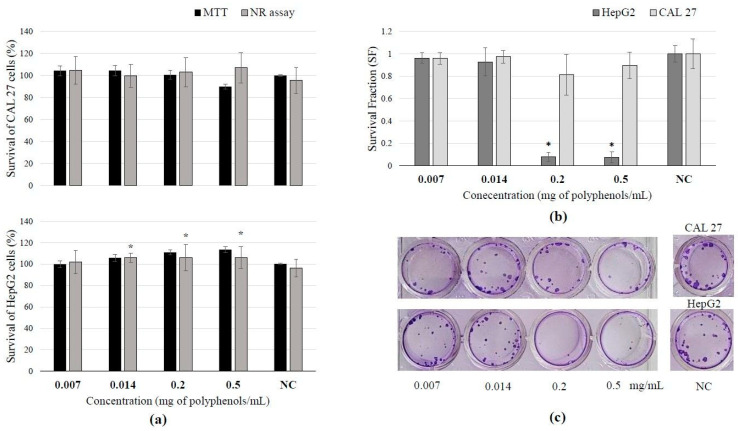
Results of BCL extract treatment on tongue epithelial carcinoma cell line (CAL 27, 30 min) and human hepatocellular carcinoma cell line (HepG2, 2 h): (**a**) cell survival measured by neutral red uptake and MTT assays; (**b**) survival fraction (SF) assessed by clonogenic assay, demonstrating reduced survival or increased sensitivity to the extract treatment; (**c**) scanned wells with colonies subjected to crystal violet and Giemsa staining after 7 days of culture. (Results are expressed as mean ± SD. * Statistically significant difference compared to the control at *p* ≤ 0.05).

The selected BCL extract obtained under optimal PLE conditions did not exhibit a cytotoxic effect on the two tested cell lines, CAL 27 and HepG2 (Figure 1a). The studied extract did not affect active transport through membranes, and no impact on membrane permeability was observed in any of the tested cell lines. In contrast, the range of tested concentrations (0.014–0.2 mg polyphenols/mL) of the extract increased the activity of mitochondrial dehydrogenases in HepG2 liver cells. This increased activity of mitochondrial dehydrogenases indicates enhanced metabolic work and energy production within the cells, which brings us to the conclusion that this extract induced proliferation of HepG2 cells right after exposure of the cells to the investigated extract. On the other hand, the highest tested concentrations of BCL extract caused a statistically significant inhibitory effect on the clonogenic growth of HepG2 cells (Figure 1b) indicating that extract actually inhibits cell division and colony formation during prolonged growth (Figure 1c).

This study used plant extract derived from the BCL; however, leaves, in general, have been underexplored in the literature. Nonetheless, leaves typically contain higher concentrations of polyphenols compared to other plant parts, suggesting that the extracts obtained from leaves may exhibit more potent biological activities [51].

Efenberger-Szmechtyk et al. [52] investigated the aqueous extract of *A. melanocarpa* obtained by 1 h of extraction in cold water. They treated colon adenocarcinoma cells (Caco-2) with concentrations ranging from 0.00034 to 0.086 mg/mL. After 72 h of incubation, it was shown that a concentration of 0.0054 mg/mL decreased cell survival by 20%. Higher concentrations increase cytotoxicity in a dose-dependent manner. Results of this study proved that in both the NR and MTT assays, there was no cytotoxic effect at any investigated concentration. This discrepancy in results can be explained by the fact that, in our experiments, cells were exposed to the extract for a much shorter time (30 min and 2 h, respectively), since it is not expected that under realistic conditions (after consumption), this extract remains longer in contact with the epithelial tissue of the digestive system. Liquids typically empty from the stomach faster than solids. A study measuring gastric emptying times found that the half-emptying time (T1/2) for a liquid meal was approximately 80.5 ± 22.1 min [53]. Also, different results can be explained by cell specificities among different cell lines used in both experiments, indicating that the same compound(s) can provoke different cellular responses.

According to the obtained results, it can be seen that BCL extract has a time-dependent antiproliferative effect. The first few hours of exposure to this extract will not show any influence on cell proliferation (Figure 1a), but if the division potency of the cells that were exposed to the extract is monitored over a prolonged time after incubation (Figure 1b), it can be noticed that a decrease in colony formation will occur in a dose-dependent manner. A similar effect was also noticed in other studies [43,52]. Owczarek et al. [43] determined that BCL did not show any antiproliferative effect on colon adenocarcinoma SW-480 cells after 48 h of treatment; the polyphenol concentration of 794.84 µg/mL caused 50% growth inhibition in colorectal adenocarcinoma HT-29 cells. Also, the viability of both cell lines decreased in a time- and concentration-dependent manner. In the same study, it was concluded that much lower extract concentrations during a shorter period of treatment caused the inhibition of tumor cell growth in comparison to normal colon cells. According to Gao et al. [54], *A. melanocarpa* fruit extract exhibited antiproliferative effects, suppressing the growth of HepG2 human liver cancer cells. At concentrations below 350 μg/mL, the black chokeberry extract did not exhibit cytotoxicity. Nevertheless, it inhibited cell proliferation, suggesting that the suppression of cell growth was not attributable to cytotoxic activity. This is similarly the case in this study.

### 3.5. Antioxidative and DNA Damage Protective Effect of BCL Extract Obtained Under Optimal PLE Conditions

**Figure 2:**
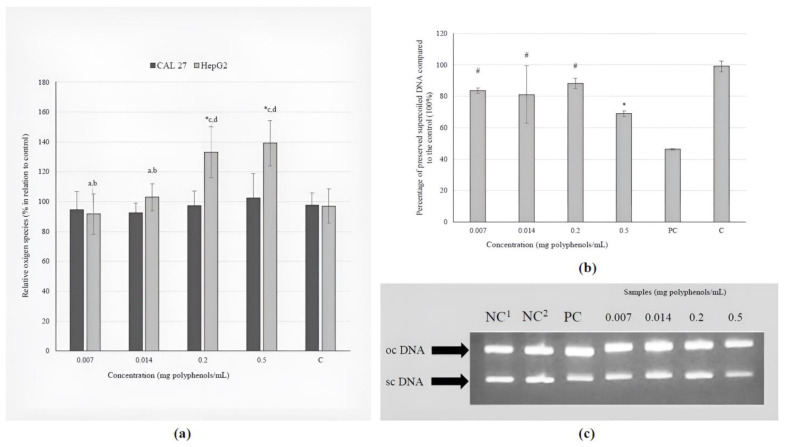
The effects of the BCL extract on: (**a**) induction of ROS in tongue epithelial carcinoma cell line (CAL 27, 30 min) and human hepatocellular carcinoma cell line (HepG2, 2 h); (**b**) oxidative DNA damage (ΦX174 RF1) generated by UV-photolysis of H_2_O_2_ (0.03 M) demonstrated as percentage of preserved supercoiled DNA. The positions of supercoiled DNA (scDNA) and open circular DNA (ocDNA) are indicated in the gel picture (**c**). Lane 1–2: negative control (NC; 1only plasmid, 2plasmid + H_2_O_2_); Lane 3: ΦX174 RF1 plasmid exposed to UV and H_2_O_2_ (positive control, PC); Lanes 4–7: ΦX174 RF1 plasmid + extract + UV + H_2_O_2_ (1–4: 0.007–0.5 mg polyphenols/mL). Statistically significant difference compared to: * NC, ^#^ PC, ^a^ 0.007, ^b^ 0.014, ^c^ 0.2, ^d^ 0.5 mg polyphenols/mL (*p* ≤ 0.05).

None of the tested concentrations of the investigated extract caused a prooxidative effect on CAL 27 cells. However, the daily recommended dose and a dose 2.5-fold higher than the recommended dose were found to have a pro-oxidation effect on metabolically active liver cells (Figure 2a).

The investigation of the direct protective effect of the BCL extract on the inhibition of hydroxyl radicals revealed that all tested concentrations, except for the highest (0.5 mg polyphenols/mL), significantly reduce damage to genetic material (Figure 2b). Different treatments create different types of plasmid DNA, which are shown by the arrows in Figure 2c. These types are oc (open circular) and sc (supercoiled).

The antioxidant effect of the BCL extract was not proven in terms of a decrease in the basal levels of free radicals in CAL 27 and HepG2 cells, but its antioxidant potency against hydroxyl radicals in a concentration-response manner was proven when a model plasmid was used. These highly reactive and consequently short-lived molecules are formed during Fenton reactions and the decomposition of hydroperoxides (ROOH) in the atmosphere [55]. Oxidative damage is an important factor in the development of various diseases, such as cardiac diseases, stroke, Alzheimer’s and Parkinson’s disease, so the discovery of chemicals that can prevent or reduce oxidative stress is of great importance.

The results of this study showed that the daily recommended dose and a dose 2.5-fold higher than the recommended dose were found to have a pro-oxidation effect on metabolically active liver cells (Figure 2a). Polyphenols are generally known for their antioxidant properties, but they can also function as prooxidants in higher concentrations. The term “hormesis” describes the biphasic dose–response relationship observed with certain chemicals, where low doses elicit a stimulatory or beneficial effect, while high doses result in an inhibitory or toxic outcome. A diet rich in vegetables and fruits is associated with numerous health benefits, including anti-aging potential, primarily due to the hormetic effects of the phytochemicals present in these plant-based foods [56]. The antioxidant activity of black chokeberry fruit has been extensively investigated; however, to the best of our knowledge, there is a lack of empirical data regarding the influence of BCL extract on %ROS in tongue and liver cancer cell lines under in vitro conditions.

The extract’s concentration range of 0.007–0.2 mg polyphenols/mL in this study demonstrated a protective effect against DNA damage generated by UV-photolysis of H_2_O_2_ (Figure 2b). Additionally, Efenberger-Szmechtyk et al. [52] reported that leaf extracts of *A. melanocarpa* (0.00034–0.00068 mg/mL) reduced DNA damage in Caco-2 cells after 60 and 120 min, with the highest efficiency of DNA repair (>80%) observed at a concentration of 0.00068 mg/mL after 120 min of incubation.

**Figure 3:**
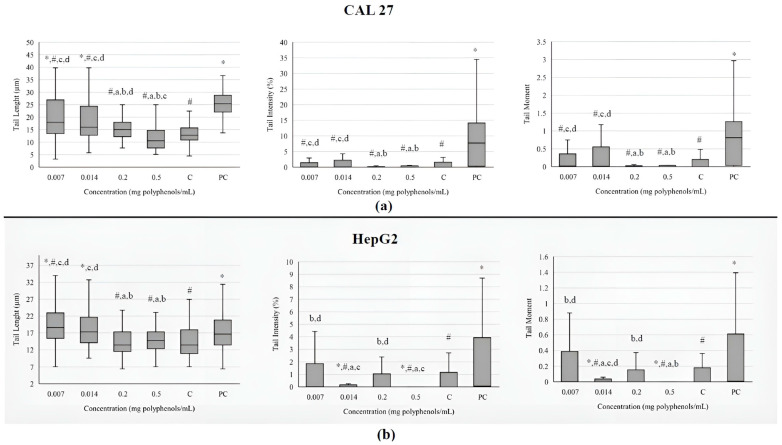
Genotoxicity on tongue epithelial carcinoma cell line CAL 27 (**a**) and human hepatocellular carcinoma cell line HepG2; and (**b**) upon treatment with the BCL extract determined by comet assay. The DNA damage is evaluated through tail length (µm), tail intensity (percentage of the DNA in the comet tail), and tail moment (tail length × % of DNA in the tail). Results are presented as the median (line), 25th and 75th percentiles (box), and range (whisker) from 100 measured cells. Statistically significant difference compared to: * negative control (C), ^#^ positive control (PC), ^a^ 0.007 mg/mL, ^b^ 0.014 mg/mL, ^c^ 0.2 mg/mL, ^d^ 0.5 mg/mL (*p* ≤ 0.05).

The investigated extract at the lowest concentrations caused an increase in the comet tail length in both cell lines (Figure 3). Still, the increased value of tail length in tongue cells was lower than the one measured in the positive control. The other two investigated parameters (tail intensity and tail moment) did not differ significantly from the negative control in CAL 27 cells. In metabolically active liver cells, tail intensity and tail moment were decreased in comparison to negative control, indicating that BCL extract has protective effect against basal damage of DNA.

The most commonly used parameters that describe the level of DNA damage in a comet assay are tail length, tail intensity and tail moment. According to some scientists, tail length is not a sufficiently reliable parameter because it only increases when tails are initially established at relatively low levels of damage. Subsequently, as the dose of damage increases, the tail intensity augments, but its length does not. Additionally, the tail measurement is sensitive to the background or threshold settings of the image analysis software (Comet Assay IVTM software, version number: Comet Assay 4.2, TE4H-V245-UXIU-KF5N, Instem-Perceptive Instruments Ltd., Bury Saint Edmunds, UK), potentially leading to excess fluorescence over the background [57]. The BCL extracts ranging from 0.0001 to 0.5 mg polyphenols/mL were found to cause statistically significant DNA damage in Caco-2 cells, as determined by the comet assay. At higher concentrations of the extracts (>0.02 mg/mL), a greater number of apoptotic cells were observed. Additionally, the maximum percentage of cells exhibiting higher levels of DNA damage increased in correlation with the extract concentration [52]. The results of this study detected a large number of highly disintegrated cells after treatment with 0.2 and 0.5 mg/mL of extract, which, due to the limitations of the comet test, cannot be quantified as measurable parameters. This may explain the observed false-negative results and discrepancies with findings from other studies.

### 3.6. Antibacterial and Antifungal Activity of BCL Extract Obtained Under Optimal PLE Conditions

Due to the high content of anthocyanins, proanthocyanidins and hydroxycinnamic acids, the antioxidant properties of chokeberry fruits are well researched, while studies on antimicrobial properties are scarce, especially the antimicrobial activity of chokeberry cultivation by-products such as leaves, twigs and pomace. In this study, the antimicrobial properties of BCL extract against Gram-positive and Gram-negative bacteria, lactic acid bacteria, yeasts and mold were determined using the well diffusion method, the microdilution method (MIC) and the colony counting method.

**Figure 4:**
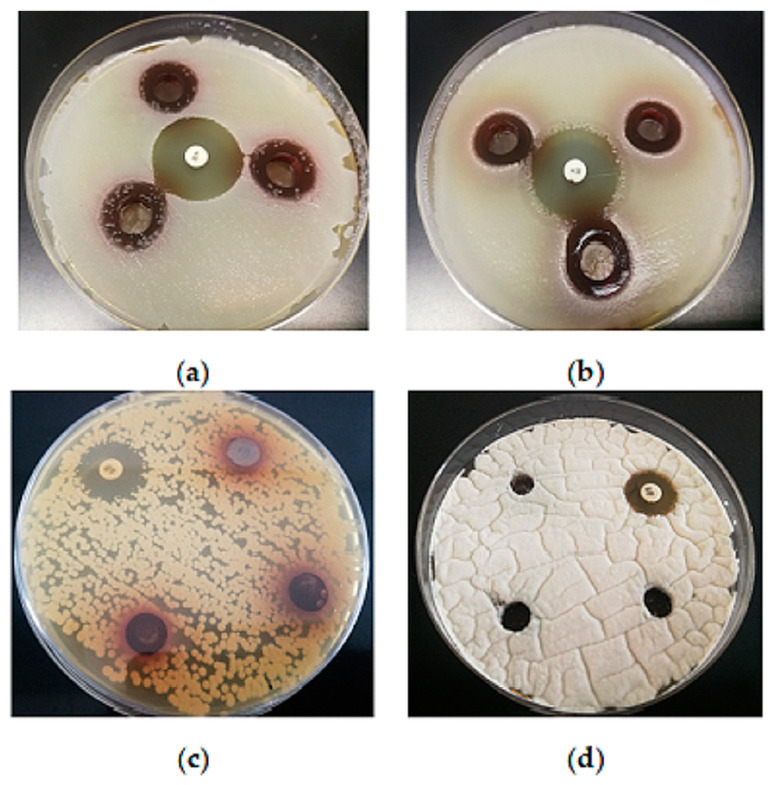
Inhibition zones of the extracts against: (**a**)—*S. aureus* (19 ± 1 mm); (**b**)—*B. subtilis* (17 ± 0 mm); (100 µL aqueous extract with a TP of 19.11 mg GAE/mL, in triplicate; K—kanamycin 50 µg disk—positive control; 26 ± 1 mm). No antimicrobial activity against (**c**) yeast *C. albicans* and (**d**) mold *Pencillium* sp.; N—nistatin—positive control (100 U); 19 ± 1 mm; 15 ± 0 mm).

Gram-positive bacteria are considered more sensitive than Gram-negative bacteria against plant extracts. This was confirmed as well in this study: the aqueous BCL extract with a TP of 19.1 mg/mL showed moderate antimicrobial activity against the Gram-positive bacteria *S. aureus* and *B. subtilis* (Figure 4), while no antimicrobial activity against Gram-negative bacteria, as well as yeasts, lactic acid bacteria and molds, was observed (Figure 4).

These results are consistent with the literature data showing that extracts from chokeberry leaves have antibacterial activity [58,59]. It was very difficult to compare the literature data because different methods are used to prepare the extracts and the content of dominant bioactive compounds varied because of this. In addition, some results refer to polyphenols’ concentration, while others are given per gram of leaf powder in the solution. However, it should be noted that Gram-negative bacteria showed the most resistance, while the chokeberry leaf extract showed antimicrobial potential against Gram-positive bacteria [59]. Denev et al. [60] demonstrated that condensed tannins are the major antimicrobial agents of the chokeberry fruits. Tannin antimicrobial activity mechanisms include inhibition of extracellular microbial enzymes, deprivation of the substrates required for microbial growth or direct action on microbial metabolism through inhibition of oxidative phosphorylation [61]. The hydrophilic extracts and proanthocyanidins isolated from the 50% EtOH extract of *A. melanocarpa* branches showed antibacterial activity against Gram-positive and Gram-negative pathogenic bacteria with MIC values that ranged from 0.04 to 3.13 mg/mL [62]. Chokeberry extract also inhibited some meat spoilage bacteria [13]. *B. cereus* was sensitive to the old leaf extracts of *A. melanocarpa*, while *E. coli* O157:H7, *S. enterica* s. Typhimurium and *Listeria innocua* were not inhibited [63]. On the other hand, Efenberger-Szmechtyk et al. [13] found no clear correlation between the influence of the plant extracts and the affiliation of the bacteria to the Gram-positive or Gram-negative groups of bacteria. The BCL extract in this study showed no antifungal activity, which is in agreement with Liepina et al. [64], who described chokeberry fruit extracts as good antibacterial agents without antifungal activity. It is possible that the fungi were less sensitive to the plant extracts due to their different cell wall structures [59]. On the other hand, Cvetanović et al. [12] confirmed the antibacterial and antifungal activity of extracts from chokeberry berries, leaves and stems obtained by subcritical water extraction and consider them a good candidate for an ingredient that can improve the shelf life of food.

Serial two-fold dilutions of the medium were used to determine the MIC of the extracts. *S. aureus* showed a slightly higher sensitivity to the extracts than *B. subtilis*. According to the literature, *S. aureus* was among the most sensitive bacteria towards black chokeberry extracts and isolated proanthocyanidins [60,62]. As it can be seen in Figure 4 and Table 5, the inhibition is significantly lower compared to the positive control, kanamycin. The number of viable cells was two decimal dilutions lower with the extract in the MIC than with the negative control. The BCL extract generally reduced µ_max_, prolonged t_lag_ and reduced log(N), depending on the type of extract, concentration and bacterial strain [13].

The antibacterial activity of the plant extract depends on the type of bacteria, the pH value of the medium and the structure of the polyphenols. Phenolic compounds are involved in the deformation and retardation of bacterial growth, inactivation of bacterial enzymes, cell wall binding and intercalation into the bacterial DNA during replication [65]. According to Taguri et al. [66], polyphenols with pyrogallol groups show higher antibacterial activity than those with catechol and resorcinol groups. The observed antimicrobial activity of the BCL extract could be attributed to the presence of phenolic compounds with pyrogallol groups, such as epicatechingallate and epigallocatechingallate, and with catechol groups, such as procyanidins, but also to the synergistic effect of all bioactive compounds in the extract.

## 4. Conclusions

In this study, the optimal conditions for the extraction of polyphenols from BCL with PLE and MAE were determined as follows: for PLE, 150 °C, 5 min static extraction time and SSR of 1:30 g/mL (TP 80.0 mg GAE/g dm) and 70 °C, and for MAE, an extraction time of 5 min and SSR of 1:30 g/mL (TP 36.4 mg GAE/g dm). The polyphenol composition of the BCL extracts obtained under the optimal conditions of both extraction procedures revealed the presence of 43 individual polyphenols, including nine phenolic acids, twenty-three flavonols, four flavones, four flavan-3-ols and three procyanidins, which, however, differed in their quantitative profile. The MAE extract of BCL contained a higher content of flavonols and phenolic acids, while the PLE extract had a higher yield of procyanidins and flavan-3-ols, demonstrating the selectivity differences of these extraction methods. However, the PLE extract from the BCL absolutely outperformed the BCL MAE extract in terms of antioxidant capacity, achieving significantly higher values in the FRAP (659.4 µmol TE/g dm), DPPH (802.2 µmol TE/g dm) and ORAC (578.9 µmol TE/g dm) assays. This BCL extract showed that not only it can protect against oxidative and DNA damage at lower concentrations, but it can also induce free radicals’ formation and DNA damage at higher concentrations, emphasizing the need for careful consideration of dosage and exposure duration in potential therapeutic applications. Moreover, it also showed a moderate antibacterial activity against Gram-positive bacteria *S. aureus* and *B. subtilis* with MIC values of 0.60 and 2.39 mg/mL, respectively.

## Figures and Tables

**Table 1:** TP in BCL extracts obtained under PLE and MAE.

Extraction Technique	Temperature (°C)	Time (min)	SSR (g/mL)	TP (mg GAE/g dm)
PLE	100	5	1:20	39.9 ± 1.1
			1:30	35.5 ± 1.3
			1:40	30.7 ± 1.0
		10	1:20	35.0 ± 0.0
			1:30	29.7 ± 0.3
			1:40	36.1 ± 0.3
	125	5	1:20	40.7 ± 0.2
			1:30	43.5 ± 0.2
			1:40	29.0 ± 1.8
		10	1:20	42.9 ± 0.8
			1:30	48.4 ± 0.4
			1:40	45.9 ± 0. 5
	150	5	1:20	35.1 ± 0.2
			1:30	80.0 ± 0.1
			1:40	64.7 ± 0.5
		10	1:20	50.8 ± 0.1
			1:30	50.8 ± 1.7
			1:40	64.2 ± 0.4
MAE	60	5	1:20	28.8 ± 0.1
			1:30	28.2 ± 0.5
			1:40	23.9 ± 0.1
		10	1:20	36.5 ± 0.3
			1:30	33.3 ± 0.8
			1:40	31.1 ± 0.4
	70	5	1:20	33.4 ± 0.7
			1:30	36.4 ± 0.6
			1:40	49.9 ± 0.5
		10	1:20	40.3 ± 0.0
			1:30	36.3 ± 0.9
			1:40	40.9 ± 0.3
	80	5	1:20	38.8 ± 1.0
			1:30	42.0 ± 2.2
			1:40	44.0 ± 0.0
		10	1:20	42.4 ± 0.5
			1:30	45.2 ± 1.8
			1:40	44.4 ± 1.1

TP = total polyphenols; BCL = black chokeberry leaves; PLE = pressurized liquid extraction; MAE = microwave-assisted extraction; SSR = sample to solvent ratio. Results are expressed as mean ± standard deviation.

**Table 2:** The influence of PLE and MAE parameters on TP of BCL extracts.

PLE		MAE	
Source of Variation	TP (mg/g dm)	Source of Variation	TP (mg GAE/g dm)
Temperature (°C)	*p* < 0.001 *	Temperature (°C)	*p* < 0.001 *
100	34.4 ± 1.1 ^a^	60	34.5 ± 1.5 ^a^
125	41.7 ± 1.9 ^ab^	70	39.4 ± 2.4 ^b^
150	57.6 ± 4.3 ^b^	80	38.4 ± 1.6 ^b^
Time (min)	*p* = 0.313	Time (min)	*p* = 0.687
5	44.3 ± 3.9 ^a^	5	37.0 ± 1.9 ^a^
10	44.8 ± 2.4 ^a^	10	37.9 ± 1.3 ^a^
SSR (g/mL)	*p* = 0.675	SSR (g/mL)	*p* < 0.001 *
1:20	40.7 ± 1.6 ^a^	1:20	30.2 ± 1.2 ^a^
1:30	48.0 ± 4.9 ^a^	1:30	39.4 ± 1.6 ^b^
1:40	45.1 ± 4.4 ^a^	1:40	42.7 ± 0.7 ^b^

PLE = pressurized liquid extraction; MAE = microwave-assisted extraction; TP = total polyphenols; BCL = black chokeberry leaves; SSR = sample to solvent ratio. Results are expressed as means ± SE. * *p* ≤ 0.05. Means with different letter within a column are significantly different at *p* ≤ 0.05.

**Table 3:** Polyphenols profile of BCL extracts obtained under optimal PLE and MAE conditions.

BCL Polyphenols	Precursor Ion (*m*/*z*)	Product Ion (*m*/*z*)	PLE (mg/g dm)	MAE (mg/g dm)
*Phenolic acids*			2.11 ± 0.13 ^a^	3.14 ± 0.19 ^b^
Quinic acid	191	127	0.01 ± 0.00 ^a^	0.01 ± 0.00 ^a^
3-*p*-caffeoylquinic acid	343	191, 169	0.06 ± 0.00 ^a^	0.08 ± 0.00 ^b^
Rosmarinic acid *	359.08	161	0.01 ± 0.00 ^a^	0.02 ± 0.00 ^a^
Chlorogenic acid *	353	191	1.92 ± 0.09 ^a^	2.95 ± 0.14 ^b^
3-*O*-ferruloylquinic acid	367	193	0.02 ± 0.00 ^a^	0.01 ± 0.00 ^a^
Caffeic acid *	179	135	0.01 ± 0.00 ^a^	0.03 ± 0.00 ^b^
Protocatechuic acid *	153	109	0.01 ± 0.00 ^a^	0.01 ± 0.00 ^a^
*p*-hydroxybenzoic acid	137	93	0.03 ± 0.00 ^b^	0.01 ± 0.00 ^a^
4,5-dicaffeoylquinic acid	515	353, 191	0.04 ± 0.00 ^a^	0.03 ± 0.00 ^a^
*Flavonols*			24.25 ± 1.27 ^a^	27.43 ± 1.64 ^b^
Myricetin *	319	273	0.12 ± 0.0 ^a^	0.11 ± 0.01 ^a^
Quercetin	303	303	3.37 ± 0.20 ^a^	3.84 ± 0.22 ^b^
Kaempferol deoxyhexoside	433	287	0.01 ± 0.00 ^a^	0.01 ± 0.00 ^a^
Kaempferol pentoside	419	287	0.01 ± 0.00 ^a^	0.02 ± 0.00 ^a^
Kaempferol	287	287	13.83 ± 0.97 ^a^	17.63 ± 1.03 ^b^
Quercetin dihexoside	627	303	1.26 ± 0.06 ^b^	0.43 ± 0.02 ^a^
Quercetin rhamnoside	449	303	0.01 ± 0.00 ^a^	0.01 ± 0.00 ^a^
Myricetin galactoside	481	319	0.06 ± 0.00 ^a^	0.11 ± 0.01 ^b^
Myricetin arabinoside	451	319	0.02 ± 0.00 ^a^	0.04 ± 0.00 ^b^
Ishorhamnetin glucoside	483	317	0.02 ± 0.00 ^a^	0.01 ± 0.00 ^a^
Myricetin rhamnoside	465	319	0.03 ± 0.00 ^a^	0.02 ± 0.00 ^a^
Quercetin vicianoside	597	434	0.01 ± 0.00 ^a^	0.01 ± 0.00 ^a^
Quercetin pentosylhexoside	597	303	1.08 ± 0.05 ^a^	1.45 ± 0.08 ^b^
Kaempferol pentosylhexoside	581	287	0.09 ± 0.00 ^b^	0.07 ± 0.00 ^a^
Quercetin-3-*O*-rutinoside *	611	303	2.68 ± 0.12 ^a^	2.27 ± 0.15 ^b^
Quercetin glucuronide	479	303	0.02 ± 0.00 ^a^	0.02 ± 0.00 ^a^
Quercetin-3-*O*-glucoside *	465	303	1.13 ± 0.08 ^b^	0.97 ± 0.06 ^a^
Kaempferol-3-*O*-rutinoside *	595	287	0.09 ± 0.00 ^b^	0.07 ± 0.00 ^a^
Quercetin pentoside	435	303	0.03 ± 0.00 ^a^	0.04 ± 0.00 ^a^
Ishorhamnetin pentosylhexoside	611	317	0.10 ± 0.00 ^a^	0.15 ± 0.01 ^b^
Ishorhamnetin hexoside	479	317	0.06 ± 0.00 ^b^	0.04 ± 0.00 ^a^
Ishorhamnetin rutinoside	625	317	0.12 ± 0.01 ^a^	0.12 ± 0.01 ^a^
Kaempferol hexoside	449	287	0.10 ± 0.00 ^b^	0.07 ± 0.00 ^a^
*Flavones*			0.10 ± 0.00 ^a^	0.08 ± 0.00 ^a^
Apigenin *	271	153	0.01 ± 0.00 ^a^	0.01 ± 0.00 ^a^
Apigenin pentoside	403	271	0.02 ± 0.00 ^a^	0.01 ± 0.00 ^a^
Luteolin rutinoside	595	287	0.01 ± 0.00 ^a^	0.01 ± 0.00 ^a^
Luteolin *	287	153	0.06 ± 0.00 ^a^	0.05 ± 0.00 ^a^
*Procyanidins and flavan-3-ols*			1.10 ± 0.08 ^b^	0.55 ± 0.02 ^a^
Catechin *	291	165, 139	0.08 ± 0.00 ^b^	0.05 ± 0.00 ^a^
Epicatechin	291	139, 123	0.21 ± 0.01 ^b^	0.10 ± 0.00 ^a^
Procyanidin B2 *	577	289	0.05 ± 0.00 ^b^	0.02 ± 0.00 ^a^
Procyanidin trimer	865	713, 575, 453	0.02 ± 0.00 ^a^	0.13 ± 0.01 ^b^
Epicatechingallate *	442.9	273, 139	0.02 ± 0.00 ^a^	0.02 ± 0.00 ^a^
Procyanidin B1	579	427, 291	0.71 ± 0.03 ^b^	0.21 ± 0.01 ^a^
Epigallocatechingallate *	459	289, 139	0.01 ± 0.00 ^a^	0.01 ± 0.00 ^a^

BCL = black chokeberry leaves; PLE = pressurized liquid extraction; MAE = microwave-assisted extraction. * Identified by authentic commercial standards. Values are expressed as mean ± SD. Means with different letter within a row are significantly different at *p* ≤ 0.05.

**Table 4:** Antioxidant capacity of BCL extracts obtained under optimal PLE and MAE conditions.

Extraction Technique	Antioxidant Capacity (µmol TE/g dm)		
	FRAP	DPPH	ORAC
	*p* = 0.002 *	*p* < 0.001 *	*p* < 0.001 *
PLE	659.4 ± 6.4 ^b^	802.2 ± 0.3 ^b^	578.9 ± 1.0 ^b^
MAE	489.6 ± 2.5 ^a^	531.7 ± 0.6 ^a^	479.1 ± 1.5 ^a^

BCL = black chokeberry leaves; PLE = pressurized liquid extraction, MAE = microwave-assisted extraction. Results are expressed as mean value ± SE. * *p* ≤ 0.05. Means with different letter within a column are significantly different at *p* ≤ 0.05.

**Table 5:** MIC of the aqueous BCL extract against *S. aureus* and *B. subtilis* and the number of living cells (log CFU) after 18 h of cultivation without antimicrobial agent and in the presence of extract and kanamycin at the MIC.

Tested Microorganisms	MIC		log CFU		
	BCL Extract (mg/mL)	Kanamycin (µg/mL)	with BCL Extract in the MIC	with Kanamycin in the MIC	Without Antimicrobial Agent
*S. aureus*	0.60	1.25	7.31 ± 0.03	7.50 ± 0.02	9.11 ± 0.07
*B. subtilis*	2.39	5	6.92 ± 0.07	7.04 ± 0.04	9.04 ± 0.48

MIC = minimum inhibitory concentration. Results for log CFU are expressed as mean value ± SD.

## Data Availability

The original contributions presented in the study are included in the article material; further inquiries can be directed to the corresponding author.
